# Enhancing cassava grater design: A customer-driven approach using AHP, QFD, and TRIZ integration

**DOI:** 10.1016/j.heliyon.2024.e36167

**Published:** 2024-08-13

**Authors:** Nana Yaa Serwaah Sarpong, Joseph Oppong Akowuah, Eric Asante Amoah, Joseph Ofei Darko

**Affiliations:** a Department of Agricultural and Biosystems Engineering, Kwame Nkrumah University of Science and Technology, Kumasi, Ghana; b Department of Mechanical Engineering, Cape Coast Technical University, Cape Coast, Ghana

**Keywords:** Customer requirement, Analytic hierarchy process (AHP), Quality function deployment (QFD), Theory of inventive problem solving (TRIZ), Cassava grater, Product enhancement, Innovative product design

## Abstract

Due to consistent cassava cultivation, small-scale processing centers rely heavily on the cassava grater. However, these machines face stagnation in innovation and design evolution, leading to inefficiencies, limited capacity, and inconsistent output. Adding to these challenges is the competitive global market, demanding a focus on design enhancements. This study employs a multi-faceted approach involving the Analytic Hierarchy Process (AHP), Quality Function Deployment (QFD) and Theory of Inventive Problem Solving (TRIZ) to prioritize customer requirements, propose technically aligned solutions, and offer innovative design options for cassava graters. A total of 10 customer requirements (CR), 21 technical solutions (TS), and 63 innovative design options (IDO) were established and prioritized, aiming for easy adoption by fabricators, engineers, manufacturers, and artisans. Implementing these insights boosts cassava grater efficiency and productivity and significantly advances knowledge. This work presents a thorough scientific framework for product design, empowering local manufacturers to remain viable and relevant in the rapidly changing field of product enhancement.

## 1 Introduction

The ‘survivor or insurance crop,’ cassava is a vital staple crop for millions. It provides a valuable source of calories and income to small-scale farmers and processors [1], especially in Sub-Saharan Africa, Asia, and Latin America. Its significance lies not only in its widespread cultivation but also in its resilience against climate variability and growing application [2].

Cassava deteriorates soon after harvest, necessitating processing it into various food products to increase its shelf life and enhance its market value [3]. Processing activities involve washing, peeling, grating, drying, milling, and frying, among many more.

Advances in mechanization have seen the development of several machines to aid these processes, such as the cassava grater, which primarily reduces the freshly peeled cassava into smaller sizes for further processing [4].

The cassava grater has been reported as one of the most frequently produced food processing equipment among 33 technologies identified among fabricators of food processing equipment [5]. It is one of the oldest food processing technologies still in use since its first introduction by the French in the 1930s in the Republic of Benin (formerly Dahomey) to teach farmers how to prepare *gari* and *tapioca* for export markets [6].

During that same decade in Nigeria, local artisans introduced and modified the earlier designs from manual to mechanical power sources. Now, village smiths, artisans, welders, fabricators and mechanics have refined the mechanized graters originally made with old scrap metals to reduce the cost of production [7], and continuous effort towards its improvement is still underway [8].

In most rural habitats and small-scale processing centers [9], this machine dominates owing to its significant contribution to the standard of living of fabricators and processors. In cassava processing activities, cassava grating is an essential unit operation [10].

However, despite its critical role in food security and economic development, the cassava grater has been plagued by a lack of innovation and upgrades. With limited innovation and technology integration, cassava grater designs have remained unchanged.

These outdated locally made technologies pose challenges such as inefficiency and low throughput [11,12]. Another noteworthy issue, highlighted by Ref. [13], is the inconsistent particle sizes produced, impacting the quality of processed cassava. Some machines also come with inefficient designs, resulting in the wastage of cassava due to uneven grating and inadequate utilization [14]. Asare-Marfo reported that most graters have high maintenance and downtime [15]. [7]. In addition to the drudgery and high labor intensity, the processing conditions are generally unsanitary and unwholesome. These challenges can be avoided with better-designed equipment.

Outside these technical challenges is the recent diversity in customer requirements (CR) and purchase behavior [16], coupled with the increase in product design competition in the international market [11]. This makes exploring ways and methods to improve traditional cassava graters imperative [17], focusing future designs on newly acquired knowledge and principles.

Some researchers have made commendable efforts in designing various cassava graters, focusing on enhancing efficiency, capacity, and overall machine performance.

Nnanna et al. (2023) recently developed a modernized cassava grating machine with a high-performance rate and high-quality output by modifying the mesh surface area and using stainless steel for critical components [18].

Bello et al. (2020) addressed power failure difficulties and increased efficiency by designing a cassava grating machine with two modes of operation [19].

Using materials found locally, Yusuf et al. (2019) created a basic pedal-operated cassava grater for rural areas that achieved a grating efficiency of 90.91 % [20].

Esteves et al. (2019) constructed a motor-operated cassava grater with adjustable grating drum rotational speeds, reaching % grating efficiency of 91.56 % [13].

Oraiku et al. (2015) also developed and evaluated a double-action cassava grating machine that effectively processed 100 kg of cassava samples, with an average feed and grating time of 146 and 200 min, respectively. This machine offered high throughput rates, minimal mass loss, and promising grating and collection efficiencies, making it a valuable asset for meeting the growing demand for cassava processing [12].

Although these methods have produced insightful findings and noticeable advancements in cassava grater designs, they have mostly remained within the boundaries of traditional mechanical engineering concepts. Their research primarily employs traditional engineering methodologies, which involve sizing various components of the cassava grater, such as the hopper, shaft, discharge chute, rotary drum, and grating teeth and testing for efficiency and capacity. In addition, it is crucial to acknowledge the limited emphasis on user-centricity and a data-driven approach for advancement in new designs, which defies modern machine and product design approaches.

The success of engineering product design nowadays transitions beyond the technical features of part sizing and testing for the machine's efficiency. It depends significantly on customer needs, business success parameters, and technological acceptance, especially in food processing [21]. In designing a groundbreaking product in the current competitive market, capturing customer requirements (CR) or feedback for translation into engineering characteristics is vital since they can recommend additional features to enhance product design and sustainability [22].

Thus, in the case of advancing the design of cassava graters, a customer-driven approach is necessary [23] to prioritize the customer's needs for better integration.

Several methodologies have been embraced to elevate product design, emphasizing the fulfillment of customer needs alongside technical excellence. Key methodologies usually adopted include the AHP, QFD, and TRIZ. Integrating these methodologies presents a comprehensive and sturdy approach, ensuring a thorough consideration of CR and technical specifications in the design process [24].

Different authors have integrated these approaches in various domains in several ways. Some notable studies include Wang and Zhang's model for elderly walkers [25]. Their combination of QFD and TRIZ involves an intricate process: gathering needs from elderly users, constructing a HOQ, and strategically resolving conflicts using TRIZ.

Similarly, Wang and Xu leverage AHP and TRIZ in the design of a household food waste recycling product [26]. Firstly, criteria and sub-criteria are obtained and compared using the AHP method to determine the degree of importance of user requirements. Secondly, the TRIZ is used to find optimal solutions for the contradictions concluded by analyzing user demand.

Jia's exploration of integrating QFD and TRIZ for mechanical product innovation is particularly noteworthy [27]. This paper studies integrating QFD and TRIZ to achieve mechanical product innovation design. It is found that QFD can only obtain customer demand, not create demand, find conflict, or solve conflict, and TRIZ can solve these two problems well.

Vongvit et al. (2017) study combined Fuzzy-QFD and TRIZ to identify innovative design alternatives for product development, showcasing a methodology that weighs customer needs and identifies design alternatives [28]. A fuzzy QFD mentions that it can be used in different ways to solve many design problems with the TRIZ Methodology to identify innovative design alternatives. The first study determines the needs of customers. A fuzzy set approach effectively determines the design requirements of product development. After that, QFD identified technical requirements and correlated with TRIZ to identify innovative design alternatives.

Lin and Zhang (2022) introduced a QFD-TRIZ model for designing a health education plate for the elderly, addressing health challenges systematically [29]. The QFD-TRIZ model was introduced into the product design to address the problems of the elderly coexisting with multiple chronic diseases, poor compliance with health management and unbalanced diets. Firstly, through interviews with elderly users, the needs collected are classified from physiological and psychological perspectives. The HOQ tool is used to rank and classify the needs to obtain a matrix of design technology needs indicators, extract the main conflict transformation problems in the matrix into a TRIZ problem model, and use the strategies provided by TRIZ combined with knowledge of healthy diets to provide a basis for the design of health education plate design for the elderly.

Zhi et al. enhance ergonomics in unmanned system control station design using HOQ and TRIZ, prioritizing user requirements and solving design problems [30].

In agricultural machinery, not much of this integration approach has been seen. One paper by Putri, Sutanto, and Bifadhlih introduces an integrative QFD-TRIZ method for thresher design improvement, contributing valuable insights for agricultural machinery enhancement [31]. The process consisted of the identification of the customer needs followed by the determination of their requirements rating, the preparation of QFD Phase 1, the creation of QFD Phase 2 for technical characteristics that do not contradict the design characteristics or the selection of alternative solutions using TRIZ for both contradictory characteristics and the thresher design improvement according to the design characteristics in the final stage.

Another study focuses on optimizing intelligent agricultural harvester design using QFD and AHP, emphasizing form, function, quality, and user optimization [32]. The paper assesses the mapping relationship between quality functions and design features, determining the design framework, weight, and importance values. AHP validates QFD design factors against user evaluation data, confirming the feasibility of the proposed intelligent agricultural harvester design and offering innovative insights for similar equipment in agricultural harvesting.

The studies above provide the extent to which these approaches have been used in product design. Despite its extensive use, none has been seen in cassava graters' design. This gap in research limits the innovative advancement in the design of cassava graters that could address current design challenges. This research aims to fill this gap.

This study, therefore, seeks IDO to consider in the quest to revolutionize and advance the design of cassava graters to improve their overall efficiency with a focus on end-user inputs. The study adopts a concurrent multi-approach that integrates the AHP, Morphological chart, QFD and TRIZ to translate CR into the new designs.

Modernizing traditional cassava grater designs is essential for addressing current challenges and advancing knowledge in agricultural and industrial equipment design. This research establishes a comprehensive framework that prioritizes CR and systematically provides technical design considerations to address them. Serving as a valuable guide for researchers, fabricators, artisans, engineers, and industries, this blueprint revolutionizes cassava grater designs to improve its overall performance for the user's satisfaction. The significance of this endeavor extends to mitigating post-harvest losses, improving processing efficiency for increased yield and food security, elevating cassava product quality for enhanced marketability and economic value, empowering farmers in developing countries, aligning with Industry 4.0 trends through technological integration, and boosting overall productivity in the cassava processing industry.

## 2 Methodology

### 2.1 Integration approach

**Fig. 1:**
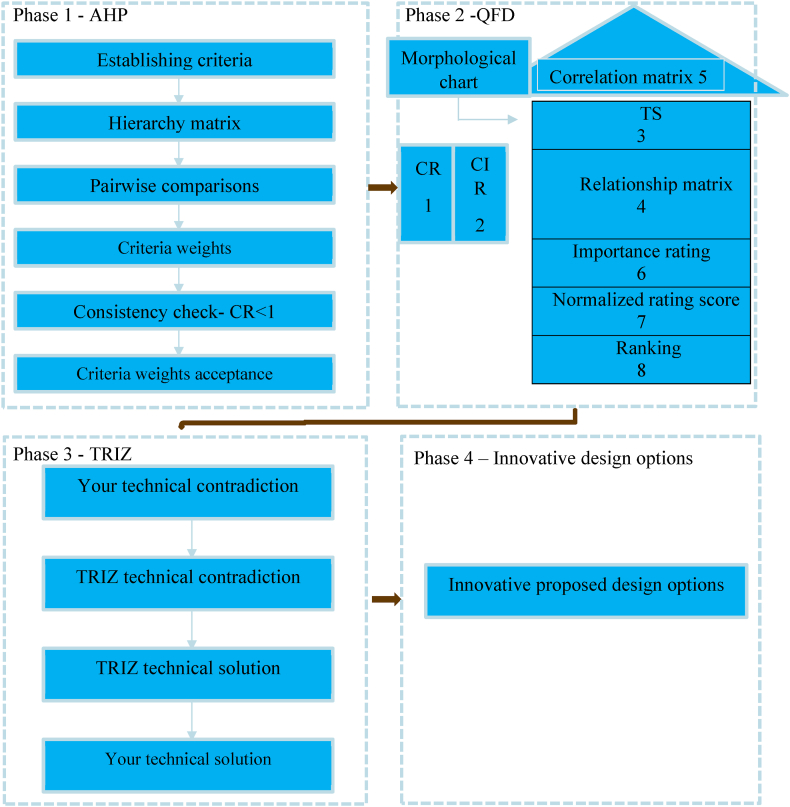
Diagrammatic representation of integration approach.

Four integral phases, AHP, QFD, TRIZ, and Product Design Options (PDO), are employed in the systematic approach to designing a new cassava grater. The integration approach is discussed in this section and a diagrammatic representation is shown in Fig. 1.

#### 2.1.1 Phase 1

In this initial stage, the AHP stage, criteria for the new cassava grater are established through a meticulous process of data collection from key stakeholders and users of the machine. The hierarchy matrix is constructed, facilitating pairwise comparisons to ascertain the relative importance of each criterion. Following the computation of criteria weights, a consistency check is performed, ensuring the reliability of the established weights.

#### 2.1.2 Phase 2

Moving to Phase 2, QFD employs a Morphological Chart and HOQ to correlate TS with CR obtained in Phase 1. The chart systematically outlines various TS, considering their relevance to customer needs. Through relationship matrices and technical importance ratings (TIR), the QFD phase establishes normalized rating scores, providing a comprehensive view of the TS effectiveness. This phase acts as a bridge between customer-centric requirements and potential technical enhancements.

#### 2.1.3 Phase 3

In the TRIZ phase, the focus shifts to resolving contradictions within the identified TS. The TRIZ process ensures that the chosen TS meets CR and overcomes inherent contradictions, fostering innovation and efficiency in the design.

#### 2.1.4 Phase 4

In this pivotal stage, the identified TS from Phases 2 and 3 are translated into actionable IDO. Each TS is analyzed to propose design modifications or enhancements that align with the resolved contradictions and CR. This step integrates adaptable mechanisms, precision engineering, and customizable features into the design blueprint. By aligning the design options with the resolved contradictions and validated TS, this phase lays the groundwork for implementing innovative and practical improvements in the cassava grater's design.

### 2.2 Description of cassava grater

**Fig. 2:**
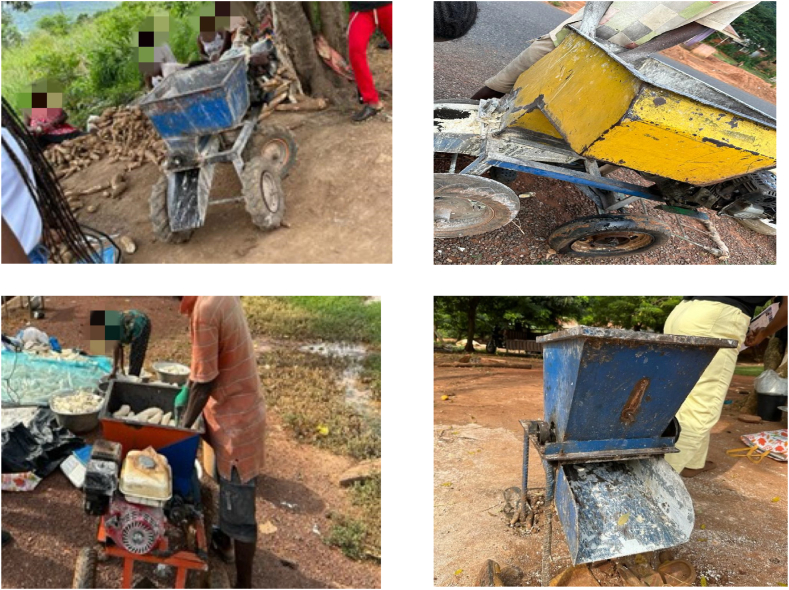
Locally made cassava grater in Ghana.

A cassava grater is a versatile machine designed for cassava processing. They come in various designs as shown in Fig. 2. It transforms raw cassava tubers into fine particles or mash. The machine comprises several integral parts, and its mode of operation involves a combination of mechanical power and precision components.

#### 2.2.1 Parts of the cassava grater

• Power source: The electric motor or engine is the primary power source, supplying the necessary rotary motion to drive the grating process.
• Transmission system: The motion and torque generated by the electric motor/engine are transmitted to the grating barrels through a well-structured transmission system. This includes pulleys, shafts, and bearings, ensuring a smooth power transfer.
• Grating barrels (drum): The grating barrels, also known as drums, are the main working components of the machine located in the grating chamber. These cylindrical drums, typically hardwood or cold-drawn mild steel, feature longitudinally milled grooves or rasping blades on the grating surface. Bearings support the barrels on either side.
• Hopper: The hopper is positioned at the top of the machine assembly, serving as the entry point for cassava tubers. Its trapezoidal design facilitates the efficient loading of raw material into the grating system.
• Discharge chute: A discharge chute is integrated into the design, slanting from the base of the hopper to guide the processed material out of the system. The chute is strategically placed to enable the flow of grated mash, typically driven by gravity.
• Frame: The entire machine assembly is supported by a sturdy frame, often constructed from angle iron. This frame provides structural integrity to the cassava grater, ensuring stability during operation.

#### 2.2.2 Mode of operation

**Fig. 3:**
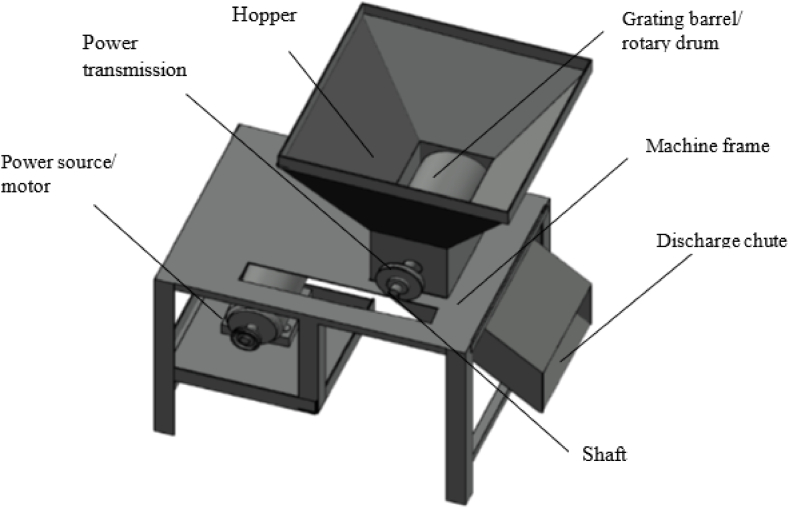
Computer aided design model of the cassava grater.

**Fig. 4:**
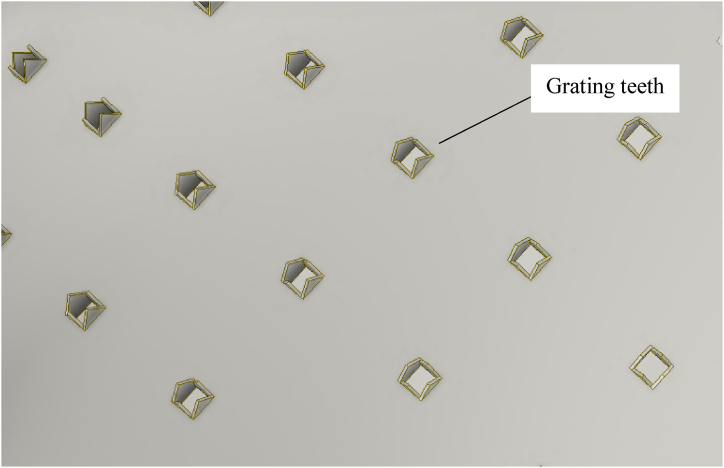
Details of a grating barrel/rotary drum.

In the cassava grating process, tubers are loaded into the top hopper of the machine as shown in Fig. 3. The grating process is initiated by the electric motor or engine, which drives the rotation of the grating barrels. As these barrels rotate, the rasping blades on their surfaces called the grating teeth as shown in Fig. 4 efficiently grate the cassava, producing fine particles. The grated material is then guided through the discharge chute, facilitated by the force of gravity, into a container placed underneath it.

### 2.3 AHP methodology for criterion prioritization

The AHP developed by Saaty (1980) is a multi-criteria decision-making tool for formulating decisions and analysis. Its primary goal is to quantify the relative importance of a given set of alternatives, criteria, or attributes on a scale of a given ratio [33] with an emphasis on the consistency of the comparison throughout the decision-making process.

The decision to employ the AHP over other methods, such as the Best-Worst Method (BWM), was based on several factors. While BWM offers advantages in capturing relative importance through the best and worst choices, AHP was selected due to its extensive application across various industries and problem domains [34]. Additionally, AHP's structured approach systematically evaluates complex criteria, offering a more comprehensive and adaptable framework for our specific research objectives [35]. Other methods, such as multi-criteria decision-making (MCDM) techniques, lack the robustness and proven versatility that AHP provides, affirming its suitability as the optimal methodology for this study.

This method's key strength is its systematic organization of concrete and intangible aspects, resulting in a structured but straightforward solution to the decision-making problem. However, AHP's subjectivity in assigning weights to criteria can introduce bias, and its reliance on pairwise comparisons may lead to inconsistency or difficulties in assigning accurate values [36]. Despite these limitations, its systematic approach and ability to blend diverse perspectives make it a valuable tool for research, especially when combined with other methodologies, contributing to informed and systematic decision-making processes. Its application is seen in many distinct sectors. This study used the AHP methodology to prioritize criteria incorporated in the grater machine design in the following steps.

Step 1 Defining clear and relevant criteria or alternatives is critical in decision-making processes. These factors should accurately represent the key aspects influencing the decision and align with the decision maker's objectives. Precise definition ensures that the criteria or alternatives considered are comprehensive, relevant, and directly contribute to the decision's outcome. This clarity minimizes ambiguity, aids in accurate evaluation, and leads to more informed and effective decisions.

Step 2 AHP is based on a pairwise comparison matrix called the Saaty Hierarchy Matrix between the number of criteria n, resulting in an nxn matrix, M, where i,j; and i=j = 1, 2, …, n.

$$M=\left(K_{IJ}\right)=\left(\begin{array}{cccc} k_{11} & k_{12} & \ldots & k_{1n}\\ k_{21} & k_{22} & \ldots & k_{2n}\\ \vdots & \vdots & \ddots & \vdots \\ k_{n1} & k_{n2} & \ldots & k_{nn} \end{array}\right)$$

On the other hand, the off-diagonal elements are reciprocal, e.g., $k_{ij}=\frac{1}{k_{ij}}\text{,}$ where $k_{ij}>0,i\neq j\text{;}$ and i,j = 1,2, …, n. The decision-maker's judgments are assigned a number based on the Saaty standard scale in Table 1.

**Table 1:** The judgment scale is based on Saaty's rating.

Scale of Importance	Definition	Explanation
1	Equal importance	Both attributes contribute equally to the objective
3	Moderately important	Judgment slightly favors one activity over the other
5	Strongly important	Judgment strongly favors one over the other
7	Very important	One activity is greatly favored over the other
9	Extremely important	There is no doubt that one attribute is of the highest validity
2,4,6,8	Intermediate values	When compromise is needed

The underlying analogy is that if an attribute or criteria A is significantly more essential than criteria B, the rating is 3, indicating that B is less important than A on a scale of 1/3. To give the given judgment more graduality, the intermediate values 2,4,6 and 8 are allocated to the adjacent scales (9,7), (7,5), (5,3), (3,1).

Step 3 Calculation of numerical weights and relevance for each rating criterion $C_{1,}C_{2,}\ldots C_{n,}$ yields a weight vector. Let, $w_{i,}$ be the weight vector, denoting the importance degree or weight for the i th attribute or criteria, then, the $w_{i,}$ is defined as follows-

w1=(∏j=1nkij)1n∑i=1n(∏j=1nkij)1n;i,j=1,2,3,…,n;andw=w1w2⋮wn

Step 4 To check the consistency and worthiness of the matrix, a consistency index C of the n column vector is calculated to take care of inconsistencies in the matrix of the pairwise comparison as follows-

$$C=\left(c_{1}\right)={K.W}_{nx1}^{T}=\begin{array}{c} c_{1}\\ c_{2}\\ \vdots \\ c_{n} \end{array},i=1,2,3,\ldots ,n$$

${A.W}^{T}$ is defined as follows-

$${A.W}^{T}=\left(\begin{array}{cccc} k_{11} & k_{12} & \ldots & k_{1n}\\ k_{21} & k_{22} & \ldots & k_{2n}\\ \vdots & \vdots & \ddots & \vdots \\ k_{n1} & k_{n2} & \ldots & k_{nn} \end{array}\right)*{\left(\begin{array}{cccc} w_{1}, & w_{2} & \ldots & w_{n} \end{array}\right)}^{T}$$

Now, the Consistency Value (CV) of the attribute might be defined by the following vector-

$$CV={\left(cvi\right)}_{nx1}=\frac{c_{i}}{w_{i}},i=1,2,3,\ldots ,n$$

As different measurement scales have been used for different attributes, Saaty suggested using the maximal eigenvalue, $\lambda_{\max}$ is defined as follows-

$$\lambda_{\max}=\frac{\sum_{i=1}^{n}{cv}_{1}}{n},i=1,2,3,\ldots ,n$$

The Consistency Index (CI) is defined as follows-

$$CI=\frac{\lambda_{\max}-n}{n-1}$$

To check the consistency of the weights, the Consistency Ratio (CR) is calculated as follows-

$$CR=\frac{CI}{RI}$$

The RI, Random Index, is a given value in the calculation process.

Step 5 The CR value must be less than 0.1 for consistent and acceptable weights. If the value of CR is less than 0.1, the weight is consistent, and the calculation process is acceptable. On the other hand, if the CR value is more significant than 0.1, then the weight is inconsistent, so the calculation process must be repeated by redefining the value of the attributes. In this approach, AHP is specifically used to express the relative preference between the rating criteria.

### 2.4 QFD for engineering solution approach

The QFD is a popular way of incorporating client wants into product conceptions [37]. QFD is a systematic product design approach involving the end-user or customer [38]. It gathers consumer feedback and incorporates it into product and technology development, refinement, and service and process standards [39]. It aims to meet customer expectations while improving product quality [40,41].

However, QFD's extensive data collection requirements and complexity can make it time-consuming, especially in cases where detailed customer input is necessary. Additionally, without robust and accurate initial customer data, QFD outputs might lack precision. Despite these challenges, QFD's emphasis on customer satisfaction and its structured approach to design prioritization make it a valuable methodology for ensuring products meet CR effectively [42].

The HOQ matrix table is commonly used as its primary tool for converting consumer demands (WHATS) into technical qualities (HOWS) [43]. The HOQ is divided into the following sections: CR obtained from the AHP process (WHATS), engineering requirements/TS (HOWS), CIR generated in the AHP, relationship matrix (HOWS vs. WHATS), correlation matrix (HOW vs. HOW), and relative importance rating (RIW). Following the receipt of the AHP results, the following actions are taken to apply this QFD.

Step 1 The average customer ratings from the AHP were computed, and 6 criteria with high averages were shortlisted and normalized to obtain their respective CIR. In effect, these ratings indicate the influence and importance of the variables to the customer.

Step 2 The next stage was to figure out how to turn the CR into TS (HOWS). 4 TS for each of the 6 shortlisted CR are provided using a morphological chart through an exhaustive review process of research publications and expert insight. These TS (HOWS) were considered with food safety, quality, and sustainability in mind.

Step 3 A QFD model is created, and experts' opinions are sorted to assign symbols and number scales for the matrices defined in Table 2 to determine how well they address and relate to the CR.

**Table 2:** HOQ judgment scales and explanations.

Symbols and numbers	Explanation
Relationship matrix	
9	Strong relationship
3	Moderate relationship
1	Weak relationship
Correlation matrix	
	Strong positive correlation
	Positive correlation
▬	Negative correlation
	Strong negative correlation
Direction of importance	
▲	Objective to minimize
▼	Objective to maximize
	Objective to hit the target

Step 4 The relative weight for each technical solution Wej is computed by multiplying the CIR of each requirement with the expert-assigned weights We and summing across all requirements for a particular TS. For j, TS and CR is-

$$Wej=\sum_{i=1}^{m}Rij\;X\;Ci,j=1\ldots .\;n\text{.}$$

Step 5 The degree of importance derived in step 4 is further normalized by dividing each element Wj of the We by the sum of all the elements. The normalized score for each technical requirement j is denoted as Wnj and yields a value in the range 1 % and 100 %

$$Wnj=\frac{wj}{\sum_{j=1}^{n}Wj},j=1\ldots .\;n\text{.}$$

Step 6 The results from the normalized score are ranked in descending order, and the TS with the highest normalized value is the best and is ranked number 1, as shown in Fig. 8.

### 2.5 TRIZ for resolving contradictions in TS

The application of TRIZ follows an initial process involving AHP for prioritizing CR and QFD for furnishing TS to address the CR. Subsequently, the technical contradictions arising from these TS are systematically addressed and resolved using TRIZ methodologies.

TRIZ, developed by G. S. Altshuller, has emerged as a powerful tool for resolving technical conflicts in innovative product design [44]. It helps create innovative designs by helping to determine the best improvement alternatives [45]. TRIZ's broad application in healthcare equipment, automotive engineering, system designs, and mechanical systems underscores its versatility and efficacy [46]. Rooted in examining a vast array of patents, TRIZ formulated 40 innovative principles linked to 39 engineering parameters and contradictions, providing a structured approach to addressing technical issues.

This method operates on the principle that problems and solutions recur across various disciplines while technical evolution patterns and scientific effects transcend their original fields [[47], [48], [49]]. TRIZ stands out due to its distinctive capability to identify problems and offer well-defined solutions, setting it apart from methods like mind mapping, morphological analysis, and lateral thinking [50,51].

The TRIZ ‘IF-THEN-BUT’ technique, as elaborated in Table 3, is adopted to understand the technical contradictions better.

**Table 3:** TRIZ ‘IF-THEN-BUT’ technique.

Term	Explanation
If	A statement where the changes are made
Then	A statement clarified the future benefits gained from the expressed action.
But	A statement explains the drawback of the expressed action.

**Table 4:** Criteria for AHP procedure.

Criteria	Notation	Definition
Performance	A1/CI	Refers to measurable operating parameters, e.g., motor speed
Reliability	A2/C2	The probability of a product surviving its design lifespan
Steady During Operations	A3/C3	Absence of noise, vibration during operation and structural stability
Aesthetics	A4/C4	Physical outlook of the product in terms of looks, color and feel
Quality Of Final Product	A5/C5	The measure of desirability of product output for food application
Repairability	A6/C6	Ease of repair and servicing during breakdown
Multifunctional Ability	A7/C7	Ability to grate different particle sizes for different food applications
Durability	A8/C8	The amount of use up to the time replacement is a better option than repair.
Energy Consumption	A9/C9	Electricity or fuel use
Affordability	A10/C10	The low initial cost of investment

Among the array of inventive principles linked to each contradiction, the selection process involves identifying one or two innovative solutions that align with the current scenario and are commonly implemented.

This deliberate selection aims to ensure that the chosen inventive principles are applicable and resonate effectively with the existing context, thereby enhancing their practical implementation within the design framework [52].

### 2.6 Proposing innovative design enhancements for cassava graters

Phase 4, the last stage after the thorough AHP, QFD, and TRIZ approaches, offers various creative solutions to improve cassava graters' design. These carefully considered options have been developed per the QFD's TS, the reconciled contradictions from TRIZ, the prioritized CR from AHP, and the analyses carried out in the preceding phases. Phase 4 is the hub where these approaches come together to offer practical and creative ideas for optimizing the design of the cassava grater for improved functionality, efficiency, and user satisfaction.

### 2.7 Data collection procedure for the various approaches

Two data sets are collected for the three techniques proposed in this research. Different approaches are used to obtain these data sets, as elaborated in this section.

#### 2.7.1 Data collection in AHP

**Fig. 5:**
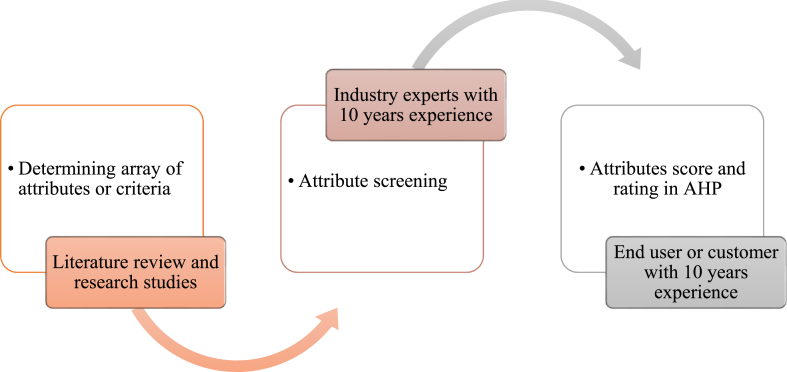
AHP data collection procedure.

The initial data set is collected for the AHP process as shown in Fig. 5. Comprehensive data collection was done in the following steps to find this study's necessary and relevant requirements.

Step 1 An extensive literature review and research studies identified various attributes specific to the advancement of cassava graters. Frequently occurring attributes were noticed and shortlisted.

Step 2 In this second step, purposive sampling was used. Professionals in the cassava grater manufacturing industry with at least 10 years of experience are interviewed to determine what clients seek when purchasing new graters. Participants who consented to the interview were presented with a questionnaire containing 39 attributes associated with cassava graters, and they were instructed to select the top 10 attributes they believe customers prioritize when making purchasing decisions. The most frequently occurring characteristics, as identified by participants' selections, were then listed. The most often occurring characteristics are listed as well. This stage drastically reduces the attributes or criteria determined in Step 1.

Step 3 This step considers the most prevalent and consistent qualities from the second step. A questionnaire based on these recurring variables is designed and distributed to a pool of cassava processors who are the end users to prioritize further and rank the attributes. When purchasing a new cassava grater, these processors are asked to select the top ten most important features using a weight scale discussed in Table 1.

#### 2.7.2 Data collection for QFD

**Fig. 6:**
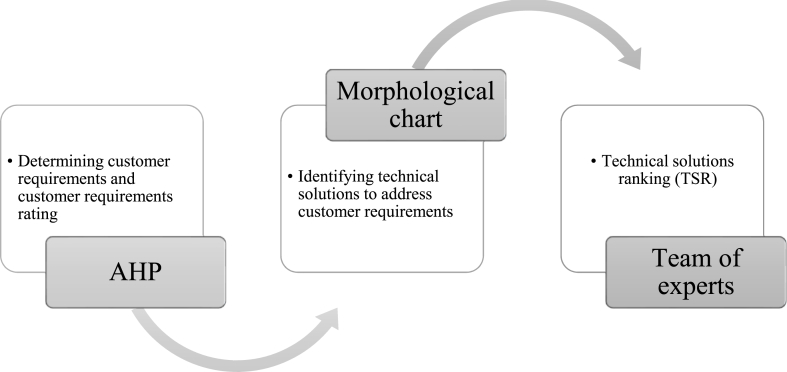
QFD data collection procedure.

The second data set is collected for this paper's second methodology, QFD. The procedure is as shown in Fig. 6. Having identified customer preference, different engineering solutions known as the HOWs that could address CR are proposed by adopting a morphological chart. For each customer's requirement, not less than 3 engineering solutions are proposed, as shown in Table 5.

**Table 5:** Morphological matrix for TS.

Morphological matrix	Technical solution 1	Technical solution 2	Technical solution 3	Technical solution 4
A1	High horsepower	Low feed rate	High machine capacity	High speed
A2	Conformance with standards	Productive working hour	Warranty	
A5	Uniformity of teeth size	Clearance between teeth edge and walls of hopper	Type of teeth (Flat/pointed/conical etc.)	
A7	Interchangeability of rotary drum	Rotary drum with varied teeth parameters	Detachable shaft	Ease of assemble and disassemble
A8	Good quality permanent and temporary joints	Corrosion resistive material	Good quality machine parts	
A9	Weight of rotary drum	Number of punched teeth	Angle or teeth arrangement	Single pass grating process

For example, energy consumption (A9) is addressed via solutions like the weight of the rotary drum, number of punched teeth, angle or teeth arrangement, and single pass grating process. These solutions target energy efficiency, optimizing the grating process while minimizing power consumption.

Based on this, the HOQ in QFD is then constructed. This model is then sent to a team of 3 experts based on their knowledge and experience of cassava graters to assign weights or scores to the relationship matrix and correlation matrix. Their response is used to calculate the importance rankings of TS.

## 3 Results and discussion

### 3.1 Customer requirements

The main objective of Phase 1 of this study was to identify CR in cassava graters with AHP. Before this, a broad spectrum of criteria was identified through an extensive literature review, and some key stakeholders identified 10 criteria to be ranked by the customers or end-users of cassava graters. These 10 attributes became the criteria and alternatives in the AHP matrix table and are prioritized based on the criteria weight assigned by customers and are defined in Table 4.

**Fig. 7:**
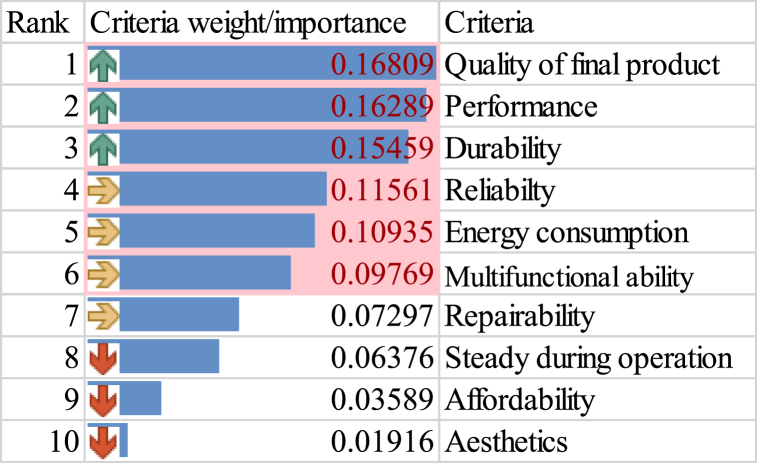
Ranked CR.

As highlighted in Fig. 7, the weight of the CR are A1-Performance (0.16289), A2-Reliability (0.11561), A3-Steady during operation (0.06375), A4-Aesthetics (0.01916), A5-Quality of the final product (0.16809), A6-Repairability (0.07297), A7-Multifunctional ability (0.09767), A8-Durability (0.15459), A9-Energy consumption (0.10935) and A10-Affordability (0.03589). Based on the prioritized weights, the most important selection factors in descending order are quality of the final product, performance, durability, energy consumption, reliability, multifunctional ability, repairability, study during operations, affordability and aesthetics.

**Fig. 8:**
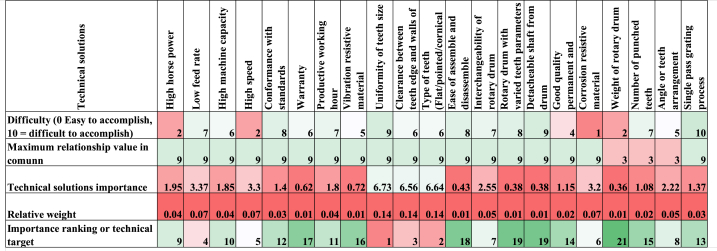
Prioritized TS and challenges.

Performance (A1) and Quality of the final product (A5) hold the highest weight among the attributes considered, indicating their significant impact on the overall design, affirming findings by Fukuda, 2009 [53]. These factors likely signify the importance of grating efficiency and output quality. Durability (A8) also holds considerable importance, suggesting that the durability of the grater is a crucial consideration for end-users. Reliability (A2) and Energy consumption (A9) follow closely, emphasizing the significance of reliable performance and energy-efficient operation in these graters. Steady operation (A3), Repairability (A6), and Multifunctional ability (A7) contribute moderately to the overall priorities, indicating that while they are essential, they might have less impact compared to other factors.

Aesthetics (A4) and Affordability (A10) have the lowest weights, indicating they might be less critical in the overall decision-making process. It's hard to believe that buyers aren't concerned with price, but in the case of cassava graters, this could suggest that processors are willing to pay any price for a machine that produces a high-quality product.

### 3.2 TS to CR

In adopting the QFD for further analysis, A1 (Performance), A2 (Reliability), A5 (Quality of Final Product), A7 (Multifunctional Ability), A8 (Durability), and A9 (Energy Consumption) were considered as the “WHATS” in the HOQ because of their weights. The TS (HOWs) that address these CR (WHATs) are obtained through the morphological chart. Food quality, safety, and sustainability were the focus while choosing these TS, as shown in Table 5.

### 3.3 Prioritizing of TS

The analysis of TS for cassava grating presents a detailed overview of challenges and priorities for manufacturers. This investigation explores these solutions' difficulty levels, importance weights, and relationship values as shown in Fig. 8. It uncovers the intricate balance manufacturers must strike between tackling critical issues and managing their complexities. Every solution has unique difficulties and significance, necessitating careful thought to balance the client's demands with functional design improvements.

The TS outlined exhibits varying degrees of difficulty, importance, weight, and relationship values crucial for manufacturers in the cassava grating industry. Notably, 'low feed rate' stands out as challenging but essential, ranking 7/10 in difficulty, yet holding a prominent 4th place in importance weight. Conversely, 'ease of assemble and disassemble' appears demanding and less critical, ranking 8/10 in difficulty but holding a lower 18th place in importance weight. This data suggests a complex trade-off between difficulty and significance that manufacturers must navigate.

Among the pivotal technical solutions (TS), 'uniformity of teeth size' and 'type of teeth' emerge as critical, ranking 9/10 and 6/10 in difficulty. However, they hold paramount importance, securing the top two positions in importance weight and ranking 1st and 2nd, respectively, in the importance ranking. This confluence of high difficulty and extreme importance indicates that these features are essential for customer satisfaction and product success despite the challenges they present to manufacturers.

For manufacturers, these findings emphasize the necessity of prioritizing efforts and resources towards addressing crucial but demanding TS, particularly those vital for customer satisfaction, such as 'uniformity of teeth size' and 'interchangeability of rotary drum.' Simultaneously, it calls for strategic decision-making when allocating resources to ensure that TS is aligned with customer needs and feasibility for implementing cassava grating processes successfully.

**Fig. 9:**
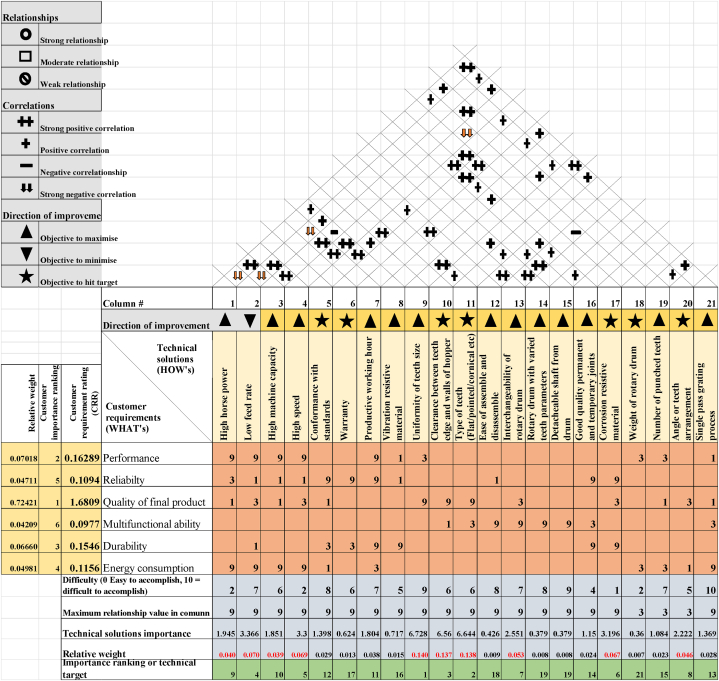
The House of Quality table by experts.

From the HOQ diagram Fig. 9., the most engineering or technical solution to be considered is determined based on the highest relative weight value reported by Ref. [54].

It is evident that the 11 most essential attributes in addressing or fulfilling the majority of customer needs in the design of a cassava grater, which translates into a product of quality value, are uniformity of the teeth size, type of teeth, clearance between teeth edge and walls of hopper, feed rate, motor speed, corrosion resistive material, interchangeability of rotary drum, angle of teeth arrangement, horsepower, machine capacity and productive working hours. All eleven highly ranked technical requirements correlated positively or negatively in the correlation matrix on the roof of HOQ.

### 3.4 Addressing technical contradictions

The technical contradictions were addressed with the TRIZ model using the correlation matrix. The TS, which yielded a strong negative correlation, is deemed contradictory. The basic principles proposed for the contradictions stemming from the correlation matrix using the 39 by 39 contradiction matrices are agreed upon as listed in Table 6. For example, if horsepower is increased, it automatically affects the feed rate, which should be as low as possible. Thus, horsepower is referred to as an improvement solution, while feed rate is referred to as a worsening solution, and the two are referred to as contradictory. In the TRIZ correlation matrix, high horsepower could be compared to Power, 21, and low feed rate to quantity of substance, 26, [55]. The matrix proposed solution to the abovementioned contradiction is solutions 4, 34, and 19, clearly defined below, with specific actions that must be taken.

**Table 6:** Application of TRIZ inventive principles.

My improvement solution	TRIZ improvement solution	TRIZ engineering parameter	My worsening solution	TRIX worsening solution	TRIZ engineering parameter	TRIZ inventive principle
High horsepower	Power	21	Low feed rate	Quantity of substance or matter	26	4, 34,19
Low feed rate	Quantity of substance or matter	26	High machine capacity	Productivity or capacity	39	13,3,27,29
High speed	Speed	9	Low feed rate	Quantity of substance or matter	26	10, 19, 29, 38
Low feed rate	Quantity of substance or matter	26	Productive working hours	Loss of time	25	35, 38, 18, 16
High speed	Speed	9	weight of the rotary drum	Weight of the mobile object	1	2,28,13,38
High machine capacity	Productivity or capacity	25	Vibration resistive material	Stability of object composition	13	35,3,22,5

Solution 4: Asymmetry.

a. Change the shape or properties of an object from symmetrical to asymmetrical.
b. Change the shape of an object to suit external asymmetries (e.g., ergonomic features).
c. If an object is asymmetrical, increase its degree of asymmetry.

Solution 34: Rejecting, Discarding - Recovering, Regeneration.

a. After completing their function (or becoming useless), reject objects, make them disappear (discard them by dissolving, evaporating, etc.) or modify them during the process.
b. Restore consumable/used-up parts of an object during operation.

Solution 19: Periodic action.

a. Instead of continuous action, use periodic or pulsating actions.
b. Change the periodic magnitude or frequency if an action is already periodic.
c. Use pauses between actions to perform a different action.

They are now considering solution 4, which states that changing the shape or property of the object from symmetrical to asymmetrical is recommended for a contradiction of horsepower and feed rate. In the case of cassava graters, the feed area, which is either a hopper or chute, could be built in such a way that the flow rate of cassava into the grating chamber is reduced. This could be achieved by exploring hopper and chute options to have as much asymmetricity as possible. For hoppers that are already asymmetric, their inner surfaces could be altered to have abrasive surfaces to increase friction, which will reduce cassava's flow rate.

### 3.5 Proposed design options to be incorporated into new designs

A systematic exploration of inventive solutions was undertaken to enhance cassava grater designs, including insight from TRIZ solutions. This approach aimed to identify innovative strategies that address specific challenges associated with cassava greater functionality and efficiency.

The analysis generated a comprehensive array of TS and design options, as shown in Table 7. These inventive solutions, derived from TRIZ methodology, provide actionable insights and design avenues to revolutionize cassava grater technology, ensuring higher productivity, operational efficiency, and durability in processing cassava tubers. For each technical solution, three innovative designs are proposed.

**Table 7:** Proposed design options.

S/n	TS	T.R	Technical improvement	Design options
1	Low feed rate	4	Adjustable feed mechanism	1 Sliding gate in hopper
				2 Adjustable entry mechanism
				3 Variable-sized entry chute
2	High speed	5	High-efficiency motor and gearing system	1 Select high-efficiency electric motor
				2 Precision gearbox for optimal speeds
				3 Use variable speed drives for control
3	High horsepower	9	Robust motor and power transmission	1 Durable motor selection
				2 Implement robust belt or chain drive
				3 Opt for reinforced gear mechanism
4	High machine capacity	10	Sturdy frame and reinforced structural components	1 Construct a frame with reinforced steel
				2 Use high-strength structural alloys
				3 Employ rigid cross-bracing
5	Productive working hour	11	Automated and streamlined processes	1 Install automated control system
				2 Implement remote start/stop features
				3 Integrate user-friendly interface
6	Weight of rotary drum	21	Lightweight drum materials without compromising strength	1 Explore advanced composite materials
				2 Use high-strength, lightweight alloys
				3 Consider reinforced composite blends
7	Interchangeability of rotary drum	7	Quick-release mechanism for Rotary	1 Implement a spring-loaded latch mechanism
				2 Use lever-operated locking pins for quick-release
				3 Employ a pneumatic or hydraulic release system
8	Ease of assemble and disassemble	18	Modular design and tool-less Fasteners	1 Design standardized interlocking modules
				2 Utilize snap-fit connectors or clip-on fasteners
				3 Incorporate twist-lock or bayonet-style connections
9	Rotary drum with varied teeth parameters	19	Customizable teeth mounting system	1 Utilize adjustable mounting brackets
				2 Implement interchangeable tooth slots
				3 Use a rail-based mounting system for teeth
10	Detachable shaft from drum	19	Quick-release shaft coupling	1 Employ spring-loaded locking collars
				2 Utilize cam-lock or bayonet couplings
				3 Design a push-button release mechanism
11	Clearance between teeth edge and walls of hopper	3	Adjustable hopper configuration	1 Implement a sliding hopper mechanism for size adjustment
				2 Utilize adjustable height settings with locking bolts
				3 Incorporate a telescopic hopper design
12	Uniformity of teeth size	1	Precision machining and quality control	1 Use CNC (Computer Numerical Control) for precise cuts
				2 Employ laser-guided machining for accuracy
				3 Implement automated inspection systems for quality checks
13	Single pass grating process	13	Optimized teeth arrangement and grating drum	1 Design helical or spiral teeth patterns for efficiency
				2 Utilize computer simulations for drum optimization
				3 Incorporate variable-depth teeth for optimal grating
14	Type of teeth (Flat/pointed/conical etc.)	2	Interchangeable teeth modules	1 Create standardized tooth modules for easy swapping
				2 Employ a snap-on modular tooth system
				3 Design a quick-release mechanism for tooth replacement
15	Number of punched teeth	15	Customizable teeth density	1 Offer interchangeable tooth inserts of various densities
				2 Implement adjustable density settings
				3 Utilize variable tooth arrangements for density control
16	Angle or teeth arrangement	8	Adjustable teeth angles	1 Incorporate a pivot system for angle adjustment
				2 Use adjustable brackets or mounts for teeth positioning
				3 Design a tooth-angle adjustment knob or lever system
17	Corrosion resistive material	6	Material selection for critical components	1 Stainless steel for durability and corrosion resistance
				2 Hardened alloys for enhanced wear resistance
				3 Food-grade polymers for lightweight yet sturdy components
18	Conformance with standards	12	Compliance with industry and safety standards	1 Adherence to ISO standards for food processing machinery
				2 Compliance with local safety regulations and certifications
				3 Design validation through third-party testing
19	Good quality permanent and temporary joints	14	Robust jointing techniques	1 Implement welded joints for structural integrity
				2 Utilize bolted connections for ease of assembly
				3 Integrate interlocking mechanisms for secure joints
20	Vibration resistive material for frame	16	Dampening and stabilization features	1 Include shock absorbers to reduce vibration and noise
				2 Design anti-skid feet for stability on different surfaces
				3 Implement rubberized components to minimize impact
21	Warranty	17	Manufacturer's assurance	1 Offer warranties for critical components and the overall product
				2 Provide technical support and maintenance guidelines
				3 Conduct quality control checks throughout the production

## 4 Conclusion

This study identified CR in cassava graters and proposed TS and design options to address them in a quest to advance and revolutionize outdated designs plagued with a lack of technological innovation. Integrating a multi-approach provided a better framework for a seamless transition from customer wants to technical hows.

The investigation into CR through the AHP surfaced 10 attributes or criteria to consider in designing and developing these machines. Performance and the quality of the final product emerged as important factors, with durability and reliability closely following, as indicated in Fig. 7.

21 TS are proposed and prioritized to satisfy these CR, highlighting manufacturers' challenges and difficulties in meeting critical customer demands using QFD.

Specific design parameters such as uniformity of the teeth size, type of teeth, clearance between teeth edge and walls of the hopper, feed rate, motor speed, corrosion resistive material, interchangeability of rotary drum, angle of teeth arrangement, horsepower, machine capacity and productive working hours standout as promising considerations engineers, fabricators, manufacturers and artisans should prioritize.

Leveraging on insight from TRIZ, 63 innovative design options are provided to address all 21 TS.

In conclusion, this study has paved a clear path toward modernizing and enhancing cassava grater designs. By effectively connecting customer needs with TS and providing innovative options, this research provides a foundation for manufacturers, engineers, and artisans to upgrade these machines.

Embracing these findings will improve the efficiency and productivity of cassava grater machines and significantly contribute to knowledge. It introduces a comprehensive methodological framework for product design, enabling local manufacturers to remain relevant and sustainable in a swiftly evolving product development landscape.

## Data availability statement

Data will be made available on request.

## Declaration of Interest's statement

The authors declare that they have no known competing interests.

## CRediT authorship contribution statement

**Nana Yaa Serwaah Sarpong:** Writing – review & editing, Writing – original draft, Methodology, Formal analysis, Data curation, Conceptualization. **Joseph Oppong Akowuah:** Writing – review & editing, Supervision. **Eric Asante Amoah:** Supervision. **Joseph Ofei Darko:** Supervision.

## Declaration of competing interest

The authors declare that they have no known competing financial interests or personal relationships that could have appeared to influence the work reported in this paper.
