# Supplementary material for: Enhancing cassava grater design: A customer-driven approach using AHP, QFD, and TRIZ integration
Source: Heliyon. 2024 Aug 13;10(16):e36167. doi: 10.1016/j.heliyon.2024.e36167 (PMC11367553; doi:10.1016/j.heliyon.2024.e36167)
Supplement: Multimedia component 1 [file mmc1.docx]

**CASSAVA GRATER MANUFACTURING INDUSTRY PROFESSIONAL INTERVIEW QUESTIONNAIRE**

**Before proceeding with the questionnaire, please read the following statement carefully:**

I understand that the information provided in response to this questionnaire will be used solely for research purposes related to the cassava grater manufacturing industry. My participation in this survey is voluntary, and I have the right to withdraw at any time without penalty. I understand that my responses will be kept confidential and anonymous, and will only be analyzed in aggregate form. By continuing with the questionnaire, I consent to participate in this research study.

**Instruction:** Please review the list of attributes provided and mark the top 10 attributes that you believe customers prioritize when purchasing a cassava grater. To indicate your selections, simply place a checkmark (√) next to each attribute you consider to be among the top 10. Thank you for your participation!

| \| 1 \| Durability \| 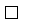   \|  \| \| --- \| \|  \| 21 \| Yield of grated cassava per batch \| 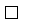   \|  \| \| --- \| \| \| --- \| --- \| --- \| --- \| --- \| --- \| --- \| --- \| --- \| \| 2 \| Efficiency \| 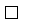 \|  \| 22 \| Affordability \| 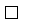 \| \| 3 \| Ease of use \| 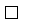 \|  \| 23 \| Brand reputation \| 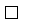 \| \| 4 \| Performance \| 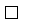 \|  \| 24 \| Warranty and after-sales service \| 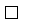 \| \| 5 \| Size and weight \| 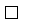 \|  \| 25 \| Reviews and recommendations \| 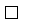 \| \| 6 \| Material quality \| 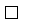 \|  \| 26 \| User-friendly controls \| 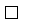 \| \| 7 \| Quality of final product \| 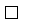 \|  \| 27 \| Repairability \| 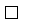 \| \| 8 \| Corrosion resistance \| 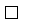 \|  \| 28 \| Capacity (batch size) \| 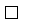 \| \| 9 \| Ergonomic design \| 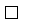 \|  \| 29 \| Compatibility with other food processing equipment \| 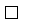 \| \| 10 \| Safety features \| 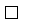 \|  \| 30 \| Customization options \| 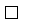 \| \| 11 \| Versatility in grating options (fine, medium, coarse) \| 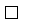 \|  \| 31 \| Multifunctional ability \| 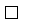 \| \| 12 \| Speed of grating \| 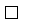 \|  \| 32 \| Space-saving design \| 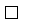 \| \| 13 \| Noise level \| 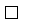 \|  \| 33 \| Resistance to wear and tear \| 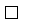 \| \| 14 \| Power source (manual, electric) \| 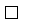 \|  \| 34 \| Aesthetics \| 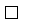 \| \| 15 \| Energy efficiency \| 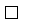 \|  \| 35 \| Environmental impact (e.g., eco-friendly materials) \| 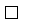 \| \| 16 \| Portability \| 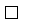 \|  \| 36 \| Availability of spare parts \| 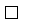 \| \| 17 \| Stability during operation \| 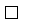 \|  \| 37 \| Compliance with safety and regulatory standards \| 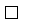 \| \| 18 \| Cleaning and maintenance requirements \| 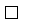 \|  \| 38 \| Reliability \| 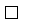 \| \| 19 \| Compatibility with different cassava varieties \| 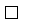 \|  \| 39 \| Steady during operation \| 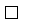 \| \| 20 \| Energy consumption \| 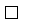 \|  \|  \|  \|  \| |  |  |  |  |  |  |
| --- | --- | --- | --- | --- | --- | --- | --- | --- | --- | --- | --- | --- | --- | --- | --- | --- | --- | --- | --- | --- | --- | --- | --- | --- | --- | --- | --- | --- | --- | --- | --- | --- | --- | --- | --- | --- | --- | --- | --- | --- | --- | --- | --- | --- | --- | --- | --- | --- | --- | --- | --- | --- | --- | --- | --- | --- | --- | --- | --- | --- | --- | --- | --- | --- | --- | --- | --- | --- | --- | --- | --- | --- | --- | --- | --- | --- | --- | --- | --- | --- | --- | --- | --- | --- | --- | --- | --- | --- | --- | --- | --- | --- | --- | --- | --- | --- | --- | --- | --- | --- | --- | --- | --- | --- | --- | --- | --- | --- | --- | --- | --- | --- | --- | --- | --- | --- | --- | --- | --- | --- | --- | --- | --- | --- | --- | --- | --- | --- | --- | --- | --- | --- | --- | --- | --- | --- | --- | --- | --- | --- | --- | --- | --- | --- | --- | --- | --- | --- |
|  |  |  |  |  |  |  |
|  |  |  |  |  |  |  |
|  |  |  |  |  |  |  |
|  |  |  |  |  |  |  |
|  |  |  |  |  |  |  |
|  |  |  |  |  |  |  |
|  |  |  |  |  |  |  |
|  |  |  |  |  |  |  |
|  |  |  |  |  |  |  |
|  |  |  |  |  |  |  |
|  |  |  |  |  |  |  |
|  |  |  |  |  |  |  |
|  |  |  |  |  |  |  |
|  |  |  |  |  |  |  |
|  |  |  |  |  |  |  |
|  |  |  |  |  |  |  |
|  |  |  |  |  |  |  |
|  |  |  |  |  |  |  |
|  |  |  |  |  |  |  |
