# Supplementary material for: Enhancing cassava grater design: A customer-driven approach using AHP, QFD, and TRIZ integration
Source: Heliyon. 2024 Aug 13;10(16):e36167. doi: 10.1016/j.heliyon.2024.e36167 (PMC11367553; doi:10.1016/j.heliyon.2024.e36167)
Supplement: Multimedia component 3 [file mmc3.docx]

AHP decision matrix by one end-user

**Fig. 9.** The House of Quality table by experts

**Fig. 8.** Prioritized TS and challenges.
